# Mitogenomic analysis of extant condor species provides insight into the molecular evolution of vultures

**DOI:** 10.1038/s41598-021-96080-6

**Published:** 2021-08-24

**Authors:** D. De Panis, S. A. Lambertucci, G. Wiemeyer, H. Dopazo, F. C. Almeida, C. J. Mazzoni, M. Gut, I. Gut, J. Padró

**Affiliations:** 1 Grupo de Investigaciones en Biología de la Conservación, INIBIOMA, Universidad Nacional del Comahue-CONICET, 8400 Bariloche, Argentina; 2 Instituto de Ecología, Genética y Evolución de Buenos Aires (IEGEBA), Universidad de Buenos Aires-CONICET, Intendente Güiraldes 2160, 1428 Ciudad Autónoma de Buenos Aires, Argentina; 3 Ecoparque Buenos Aires-Argentina, República de la India 3000, 1425 Ciudad Autónoma de Buenos Aires, Argentina; 4 Fundación Cabure-Í, Mcal Antonio Sucre 2842, 1428 Ciudad Autónoma de Buenos Aires, Argentina; 5 Berlin Center for Genomics in Biodiversity Research (BeGenDiv), Königin-Luise-Straße 6-8, 14195 Berlin, Germany; 6 CNAG-CRG, Centre for Genomic Regulation (CRG), Barcelona Institute of Science and Technology (BIST), Baldiri i Reixac 4, 08028 Barcelona, Spain; 7 Present Address: Hospital Escuela, Facultad de Ciencias Veterinarias, Universidad de Buenos Aires, Av. Chorroarín 280, 1427 Ciudad Autónoma de Buenos Aires, Argentina

**Keywords:** Evolutionary genetics, Comparative genomics, Conservation genomics

## Abstract

The evolution of large vultures linked to mountainous habitats was accompanied by extreme physiological and behavioral specializations for energetically efficient flights. However, little is known on the genetic traits associated with the evolution of these obligate soaring scavengers. Mitochondrial DNA plays a vital role in regulating oxidative stress and energy production, and hence may be an important target of selection for flight performance. Herein, we characterized the first mitogenomes of the Andean and California condors, the world’s heaviest flying birds and the only living representatives of the *Vultur* and *Gymnogyps* genus. We reconstructed the phylogenetic relationships and evaluated possible footprints of convergent evolution associated to the life-history traits and distributional range of vultures. Our phylogenomic analyses supported the independent evolution of vultures, with the origin of Cathartidae in the early Paleogene (~ 61 Mya), and estimated the radiation of extant condors during the late Miocene (~ 11 Mya). Selection analyses indicated that vultures exhibit signals of relaxation of purifying selection relative to other accipitrimorph raptors, possibly indicating the degeneration of flapping flight ability. Overall, our results suggest that the extreme specialization of vultures for efficient soaring flight has compensated the evolution of large body sizes mitigating the selection pressure on mtDNA.

## Introduction

Identification of the processes influencing genetic variation is critical to understand complex phylogenetic patterns, and essential for an accurate assessment of the conservation status of threatened species^1–3^. Previous studies revealed that the high aerobic capacity of the flight muscles of birds is modulated through the oxidative phosphorylation capacity of the mitochondria^4^. Thus, mtDNA may be an important target of natural selection, especially in high altitude birds exposed to cold temperatures and low oxygen pressure. For instance, selective analyses in Andean sparrows (Z*onotrichia capensis*) showed an elevational cline related to mt-haplotype frequency, but not with nuclear markers^5^, while a single mtDNA mutation was found to contribute to the exceptional ability of the bar-headed geese (*Anser indicu*s) to migrate over the Himalayas^4^ and recent mitogenomic analysis revealed evidence of polygenic selection related to mitochondrial efficiency in high altitude Galliforms^6^. Despite the extreme specialization for highly efficient flights and the fact that many vultures inhabit high mountain areas, the role of mitochondrial adaptation in this guild remains unexplored.

Vultures constitute the only obligate vertebrate scavengers, exhibiting a wide array of adaptations that allows them to cover vast territories in search of patchy and unpredictable carrion resources^7^. Given their striking similarities, the phylogenetic relationships of this group have been challenging for many years, traditionally placing New World (NW) vultures (Cathartidae) along with Old World (OW) vultures (Accipitridae) in the order Falconiformes or in their own order^7,8^. Moreover, relationships within Cathartidae are still unclear largely due to incomplete taxonomic sampling and limited genetic resolution^9^. Recent nuclear genomic analyses showed that Falconiformes excluded the Turkey vulture (*Cathartes aura*), and suggested that Cathartidae split from a common ancestor of their sister group Accipitridae at some point between the late Cretaceous and early Paleogene or even later^10,11^. However, nuclear genomic data is still scarce and the inclusion of more taxa is needed to test this hypothesis and to provide more precise dating. The use of mitogenomic data, on the other hand, has provided increased taxon sampling, including several OW vultures, resulting in powerful phylogenetic inferences^12–14^. However, with only one species annotated, NW vultures are still underrepresented in their availability of mitogenomic resources, leading to their exclusion from phylogenomic studies due to long branch attraction problems^12,15,16^.

The two extant species of condors represent both the most iconic and threatened vultures of the New World. The Andean (*Vultur gryphus;* wingspan of 3.2 m and weigh up to 16 kg) and California condor (*Gymnogyps californianus;* wingspan of 2.9 m and weigh up to 14 kg), are the World’s heaviest flying birds and the only living representatives of their genus^7,17^. While the latter has been the subject of intense, and so far successful, conservation efforts to prevent its extinction through the use of reproduction programs^3,18,19^, the former continues in steep decline. Recently, the Andean condor has been classified globally as Vulnerable and is Critically Endangered in their northern distribution due to persistent human persecution^19,20^. Previous analysis using historical samples of California condors from the nineteenth century revealed that the remarkable low levels of mtDNA diversity found in extant individuals are a direct consequence of recent human action^18^. Unlike the California condor, however, recent studies of the Andean condor including museum samples collected across their historical range almost two centuries ago revealed that although this species lost significant genetic variation during the early twentieth century, low mtDNA diversity is mostly ancient^2^. Notwithstanding, Andean condors from the southern Andes still retain normal levels of nuclear genetic variation^21,22^, suggesting that evolutionary constraints on genetic diversity only affected the mitochondrial genome.

It has been argued that the evolution of the body mass of vultures reflects the physiological limits imposed by the flight conditions in each habitat. Thus, the largest vultures occur in mountainous landscapes where the intense winds provide strong updraughts, while smaller ones inhabit lowlands with weak kinetic energy^23,24^. Condors, for example, are highly dependent on high mountain slopes to soar and are predicted to experience one of the highest metabolic costs of flapping flight^25^. California condors can fly > 1.000 m above ground level in the mountains of central California (up to 3.000 masl), while Andean condors can fly > 5.000 masl across the high Andes, and both species can move 350 km in a single day^26–28^. In addition, male condors dominate over females, possibly forcing them to schedule poorly efficient routines, resulting in higher selective pressures for metabolic efficiency in the latter^17^. Indeed, female Andean condors fly at higher altitudes^28^, and experience greater physiological costs associated with longer flights, resulting in shorter telomere lengths than males^29^. Similarly, the Himalayan Griffon (*Gyps Himalayensis;* wingspan of 2.89 m and weigh up to 12 kg) and the Cinereus vulture (*Aegypius monachus:* wingspan of 3 m and weigh up to 13 kg), which might be considered as condor’s OW counterparts due to its body size and mountainous distribution, experience important selection pressures related to flight performance. The Himalayan Griffon, for example, can fly at extremely high elevations of up to 6.500 masl, employing behavioral adaptations^30^, while the Cinereus vulture exhibit a specialized hemoglobin alpha^D^ subunit of high oxygen affinity^31^. However, whether flight adaptations of large vultures involved the selection of mitochondrial variants is unknown.

Herein, we characterized the first complete mitochondrial genomes of the Andean and California condor. We investigated the phylogenetic relationships and divergence times across raptor species and evaluated whether the observed low mtDNA diversity in the Andean condor is the result of genetic constraints or a lack of characterization of informative mt-loci. We also performed selection analyses in the mitogenome of large species of NW and OW vultures to assess possible footprints of convergent evolution associated to their extreme life-history traits and mountainous distribution.

## Methods

### Sampling and sequencing

Total DNA was extracted from blood cell samples from a captive male Andean condor (ID: 01C5-218A), used as a primary genetic founder in the breeding program of the Buenos Aires Zoo (Ecoparque BA). The individual was originally from the Andean province of Mendoza (Argentina), a region encompassing the highest mountains in the Americas (up to 6.961 masl). We followed a standard phenol:chloroform extraction protocol for high-molecular-weight DNA and sequenced using a combination of short and long reads, employing the Illumina HiSeq4000 platform (2 × 151 bp, insert size = 350 bp) and Oxford Nanopore Technology MinION (two flow-cells; mean length: ~ 10 kbp) at the Centre Nacional d'Anàlisi Genòmica (Barcelona, Spain). We mapped our sequences against the reference mitogenome of the Turkey vulture and chicken *Gallus gallus* (Table S1), resulting in 13.195 paired-end short reads and 60 long reads with an approximate coverage of 234 × and 36 × , respectively. In addition, we also mapped the WGS Illumina dataset of the California condor^3^ (NCBI accession: SRR14067635), obtaining 26.790 paired-end reads (2 × 150 bp) and a coverage of 472 × .

### Assembly, annotation and comparative analysis

To assemble our mitochondrial sequences from de novo, we employed a combined strategy using alternative approaches. We used NOVOPlasty 4.2^32^, seeded with the longest mitochondrial sequence available for each species (Andean condor: 2671 bp, Accession: AF173575.1; California condor: 2672 bp, Accession: AF173574), the multiple-seeded GetOrganelle 1.7.1^33^ to include the partial sequence of their control region (Andean condor: 501 bp, Accession: AY129644.1; California condor: 569 bp, Accession: KX379719) and the uninformed method of UniCycler 0.4.8^34^, which takes advantage of the long reads in combination with the short reads of the Andean condor. Given the reported conserved gene order between Andean condors and chickens^35^, we aligned our sequences to the reference mitogenome of *G. gallus* in addition to *C. aura*, resulting in coherent consensus sequences. The assemblies were annotated with MITOS 2^36^ using the vertebrate genetic code and RefSeq 89 Metazoa as reference and visualized with OGDRAW 1.3.1^37^. All genes (tRNAs, rRNAs and PCGs), as well as the control region (CR) were manually curated. We compared the features of our assembled mitogenomes with all available vultures (*C. aura, G. fulvus, G. himalayensis, G. coprotheres* and *A. monachus*), including nucleotide identity similarity and relative amino-acid and synonymous codon usage. In addition, we examined CG/AT skew rates, analyzed tRNAs secondary structures, and assessed the characteristics of the CR (see details in Supplementary Material).

### Phylogenetic analysis and molecular clock

To determine the phylogenetic position of NW vultures, we used our assembled mitogenomes along with other 35 species/genera of birds available in the GenBank database (Table S1): 26 representative taxons of raptors (Accipitridae, Cathartidae, Falconidae, Pandionidae, Sagittariidae and Strigidae), and 9 outgroup species comprising Galliformes and Anseriformes. We used PhyloSuite v1.2^38^ to conduct the bioinformatic pipeline with the help of several programs. The annotation of the mitogenomes was standardized and ambiguously annotated tRNA genes were predicted with ARWEN v1.2.3.c^39^. All sequences were aligned with MAFFT v7.487^40^ (using the codon and normal mode for PCGs and RNAs, respectively) and PCG alignments were further refined with MACSE v2.0^41^. Ambiguously aligned fragments were removed in batches using GBLOCKS v0.91^42^ with a minimum length of a block of ten and allowing gap positions. We used MODELFINDER^43^ to find the best partition schemes and evolutionary models, employing the corrected Akaike information criterion and unlinked branch lengths. To inspect for possible sources of phylogenomic disagreement, we analyzed our trees using three predefined schemes: (1) 13 PCGs; (2) 13 PCGs + 2 tRNAs and; (3) 13 PCGs + 2 rRNAs + 22 tRNAs. To construct the phylogenetic trees, we employed maximum likelihood (ML) methods using IQ-TREE v1.6^44^ with 10.000 ultrafast bootstraps and the Bayesian inference (BI) analysis of MRBAYES v3.2.2^45^, using two simultaneous runs with one cold and three heated chains and 50 × 10^6^ MCMC repetitions (25% burn-in period), sampling every 1.000 generations until stationary distribution was reached (i.e., average standard deviation of split frequencies < 0.01).

We also estimated the divergence times to further explore the temporal frame of the phylogenetic radiation of NW vultures. For this, we used our best mitogenomic scheme (13 PCGs + 12S and 16S; see Results) and employed the Bayesian approach implemented in BEAST2^46^, assuming a relaxed log-normal clock and the Yule speciation model. Substitution and clock models were unlinked to allow independent estimation of parameters for each partition (noncoding/coding and codon positions), which were analyzed under the GTR substitution model with a four-category gamma distribution of rate variation among sites. To calibrate the molecular clock, we employed three calibration nodes (constrained to be monophyletic) based on the probability distributions of clade age from fossil occurrences. Following previous studies^47^, we set priors with log-normal distributions for the age of the clade Galloanseres (offset = 66.0, μ = 1.9, σ = 1.1), the clade uniting Anseranatidae/Anatidae (offset = 66.0, μ = 1.5, σ = 1.0) and Pandionidae/Accipitridae (offset = 33.9, μ = 1.7, σ = 0.8). Given the controversial and limited evidence of fossil records for Cathartidae^9^, we refrained from using calibration constraints on this clade. Our Bayesian inference was determined by two independent runs using 10 × 10^8^ MCMC steps, sampling every 1000 generations. The evaluation of MCMC convergence was estimated in Tracer 1.7^48^ and considered acceptable when the effective sample size of all parameters reached > 200. The maximum credibility tree was generated in TreeAnnotator v2^46^, combining all independent runs after excluding a 25% burn-in period.

### Selection analysis

We assessed signals of selection footprints in the PCGs by estimating the nonsynonymous-to-synonymous substitution rate ratio (ω = dN/dS). For this, we inferred a phylogenetic tree (as previously described) including the available sequences from all raptor species included in the Afroaves clade (Table S1). We used EasyCodeML 1.31^49^, employing the codon-aware filtered gene alignments to fit the models to our data. Following recent studies^50^, we first performed an exploratory analysis estimating ω values for each terminal branch using the free-ratios Model 1 to discard extreme results due to high saturation or low substitutions values. Because mt-genes tend to be conservative, we compared the fit of the Clade model C (CmC) with Likelihood Ratio Tests (LRT) in order to detect subtle differences in site-specific selective constraints across the phylogeny^51^. Our comparisons included: (A) vultures and the rest of the Accipitrimorphae; (B) NW and OW vultures; (C) All large and small-sized vultures; (D) Large and small-sized species within OW vultures; and (E) within NW vultures (see Fig. 4). We also performed correlation analyses between the maximum reported weight of each vulture and our ω estimations for all PCGs. To further explore the data, we also tested the fitting of equivalent Branch models (BM) and Branch-Site models (BSM, one species at a time) for genes exhibiting LRT *P-*values < 0.1 in the Clade model C analysis (see details in Supplementary Material).

## Results and discussion

### Mitogenomic architecture

**Figure 1:**
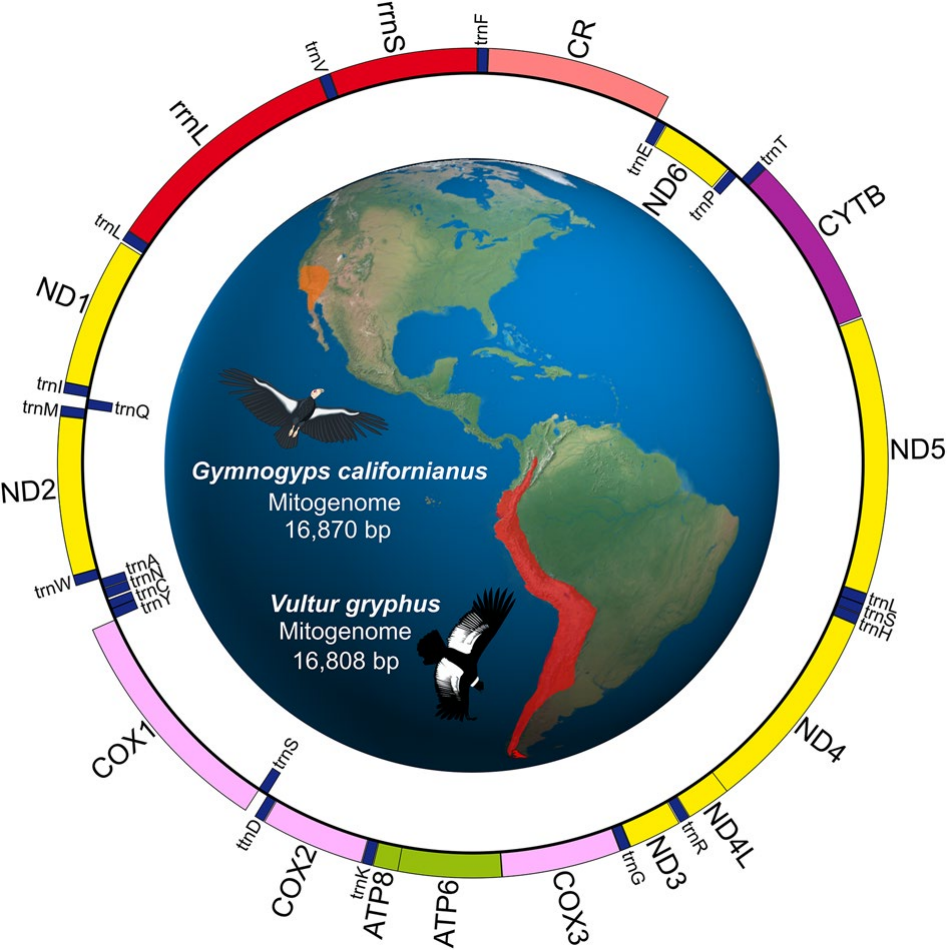
Organization of the mitochondrial genome of the Andean and California condor (identical schemes were recovered for both species; genes situated on the H and L strands are denoted in the outer and inner ring, respectively). The distributional range of both condor species is shown on the map. Mitogenomic assembly was schematized with OGDRAW 1.3.1 (https://chlorobox.mpimp-golm.mpg.de/OGDraw.html). Condor icons were taken and modified from www.freepng.es, while the world map was taken and modified from http://commons.wikimedia.org, licensed under Creative Commons Attribution 4.0 Unported license (http://creativecommons.org/licenses/by/4.0/).

**Figure 2:**
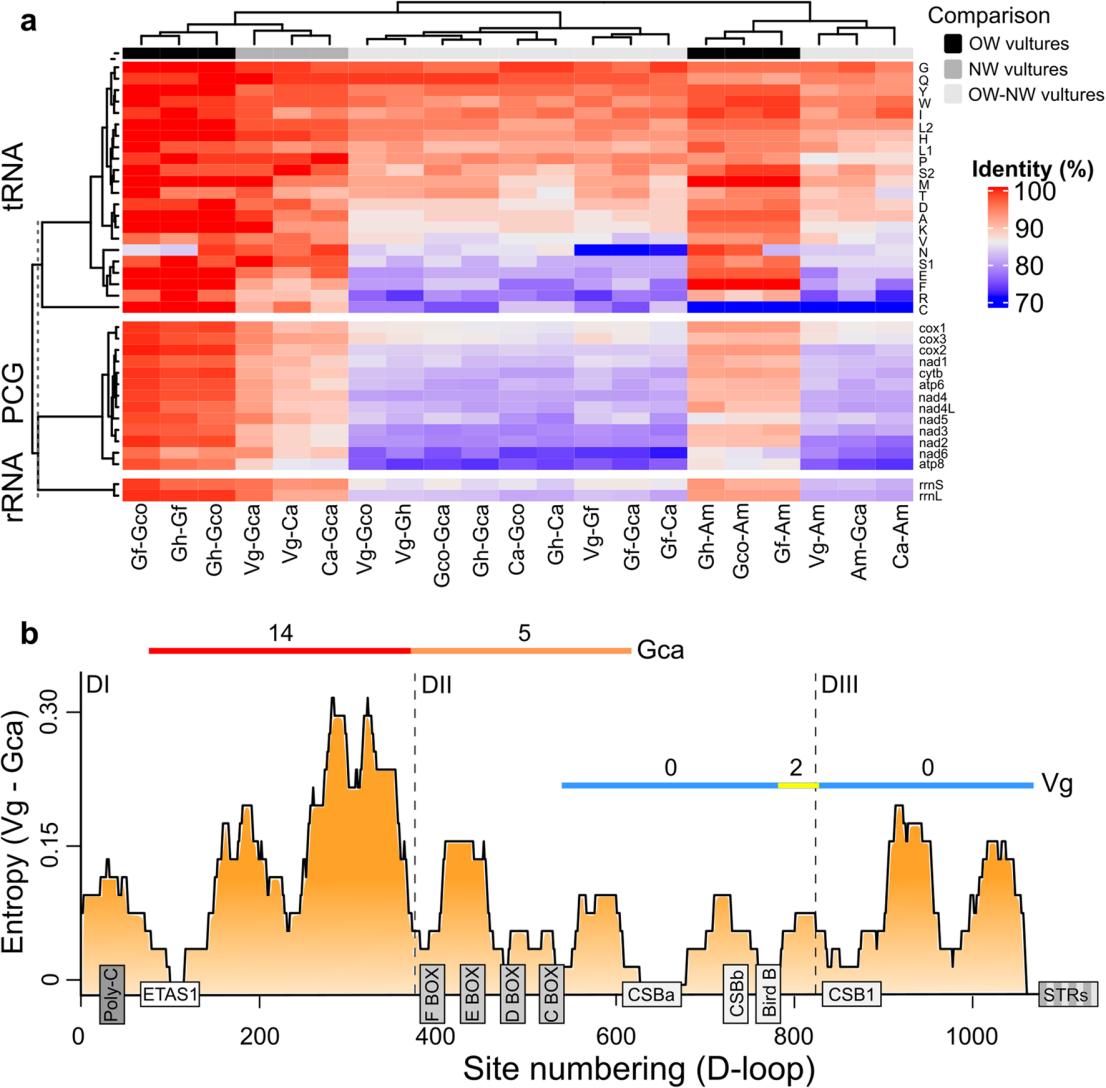
(**A**) Heatmap showing genetic identity of mitochondrial genes within and between New and Old world vultures. (**B**) Variable substitution rates over sites (entropy) between the Andean and California condor D-loop region, based on a 50-bp sliding window (interspecific variation). The organization of conserved elements and domain limits are denoted. Cold and warm colored bars indicate invariant to highly variable regions reported for both species of condors (intraspecific variation: the number of polymorphic sites is indicated at the top). Vg: *V. gryphus*; Gca: *G. californianus*; Ca: *C. aura*; Gh: *G. himalayensis*; Gf: *G. fulvus*; Gco: *G. coprotheres*; Am: *A. monachus*. Heatmap and entropy charts were plotted with R 4.0.2 (https://www.r-project.org).

The mitochondrial genome of the Andean and California condor was 16.808 and 16.870 bp in length, respectively, containing all 37 genes typical of vertebrates, including 22 tRNAs, 2 rRNAs, 13 PCGs, and a control region (Fig. 1). As previously observed in partial mtDNA sequences^35^, NW vultures exhibited the ancestral avian gene order, while OW species differed from the standard arrangement due to the presence of a pseudo control region^14,52–54^. The overall base composition of condors was similar (A =  ~31%, T =  ~23%, C =  ~32%, G =  ~13%), showing a slight A + T bias for most regions, except *ND6* that showed a marked C + G bias, similar (*r* > 0.9; *P* < 0.001 in all cases) to other vultures (Figure S1). Our comparative identity analysis revealed a clear phylogenetic pattern for both PCGs and rRNAs, except for *COX1* and *COX3* that showed a relatively high similarity between OW and NW vultures, suggesting an important evolutionary constraint acting on these regions. Most tRNA sequences were highly conserved across species, except for *trnR, S1, E, F, N* and *C*. The latter two showed an unusually high divergence, even within OW vultures, suggesting a possible pattern of selective relaxation (Fig. 2a). The tRNAs of condors displayed the typical cloverleaf of secondary structures found in other vultures, exhibiting larger structural differences with OW vultures (Figure S2).

Our analysis of PCGs in condors showed that *COX3*, *ND4* and *CYTB*, share the same transcriptional exceptions of Turkey vultures, where TAA stop codon is completed by the addition of two 3' A residues to the mRNA. This exception has been reported for *COX3, ND2* and *ND4* in the Griffon (*Gyps fulvus*) and Cape (*Gyps coprotheres*) vultures^53,54^, and for *ND2* in the Cinereous vulture and Himalayan griffon^14,52^. In addition, we found a frameshift in the *ND3* gene of both condors, suggesting an alternative reading of mitochondrial mRNA. Previous studies have found that this feature is present in some birds, including some *Cathartes* species, the Cape and Griffon vultures, but seems to be absent in the Himalayan Griffon and Cinereous vultures, suggesting the existence of additional evolutionary constraints in the former taxa due to the maintenance of a secondary structure in the mRNA^55^. Overall, we found that the relative amino-acid and synonymous codon usage in condors was similar to other vultures, exhibiting a frequent use of Leu, Thr, Ala, Ile, Gly, Ser, and Pro, with a common codon usage of CUA, ACA, GCC, AUC, GGA, UCA, and CCA, respectively (Figure S3).

The length of the CR of the Andean condor (1214 bp) was similar to the California condor (1260), Turkey vulture (1177 bp) and *Gyps* species (1201–1206 bp; *A. monachus* was excluded due to the poorly resolved CR). Our finding of high similarity of the Andean condor CR with that of the California condor (90%), Turkey vulture (88%) and *Gyps* species (~ 68%), similar to the observed in PCGs (Fig. 2a), is remarkable given that the CR of birds tends to evolve faster than PCGs^56^. We detected common conserved regions in all three domains, following the same order in all vultures (Fig. 2b; Table S2). In the first domain, we found a Poly-C region usually observed in raptors^14^, but exhibiting a TA island in the Andean condor, California condor, Turkey vulture and the Griffon vulture, and we also identified the adjacent extended termination-associated sequence ETAS1. The central domain contained short conserved blocks F, E, D, C and conserved sequence blocks CSBa, CSBb and the avian-specific Bird Box, while the third domain included CSB1 (Fig. 2b). However, we did not detect the presence of ETAS2, CSB2 or CSB3 in any species, suggesting that these sequences are not conserved among vultures. We detected a defined Short Tandem Repeat sequence (motif: AACAAAC) at the 3’ end of the third domain (Fig. 2b), in the Turkey vulture (14 repeats), the California (24 repeats) and Andean condor (20 repeats), previously reported as a source of length heteroplasmy in the latter^35^. Notably, this pattern is not present in OW vultures, where repeats are rich in T, and are located in the pseudo control region^14,52,53^. The STRs of the Turkey vulture was able to form a stable stem-loop secondary structure, similar to those reported in some Strigiforms^57^, while the stem of both condors was found in the terminal region of the third domain adjacent to the STRs (Figure S4).

**Figure 3:**
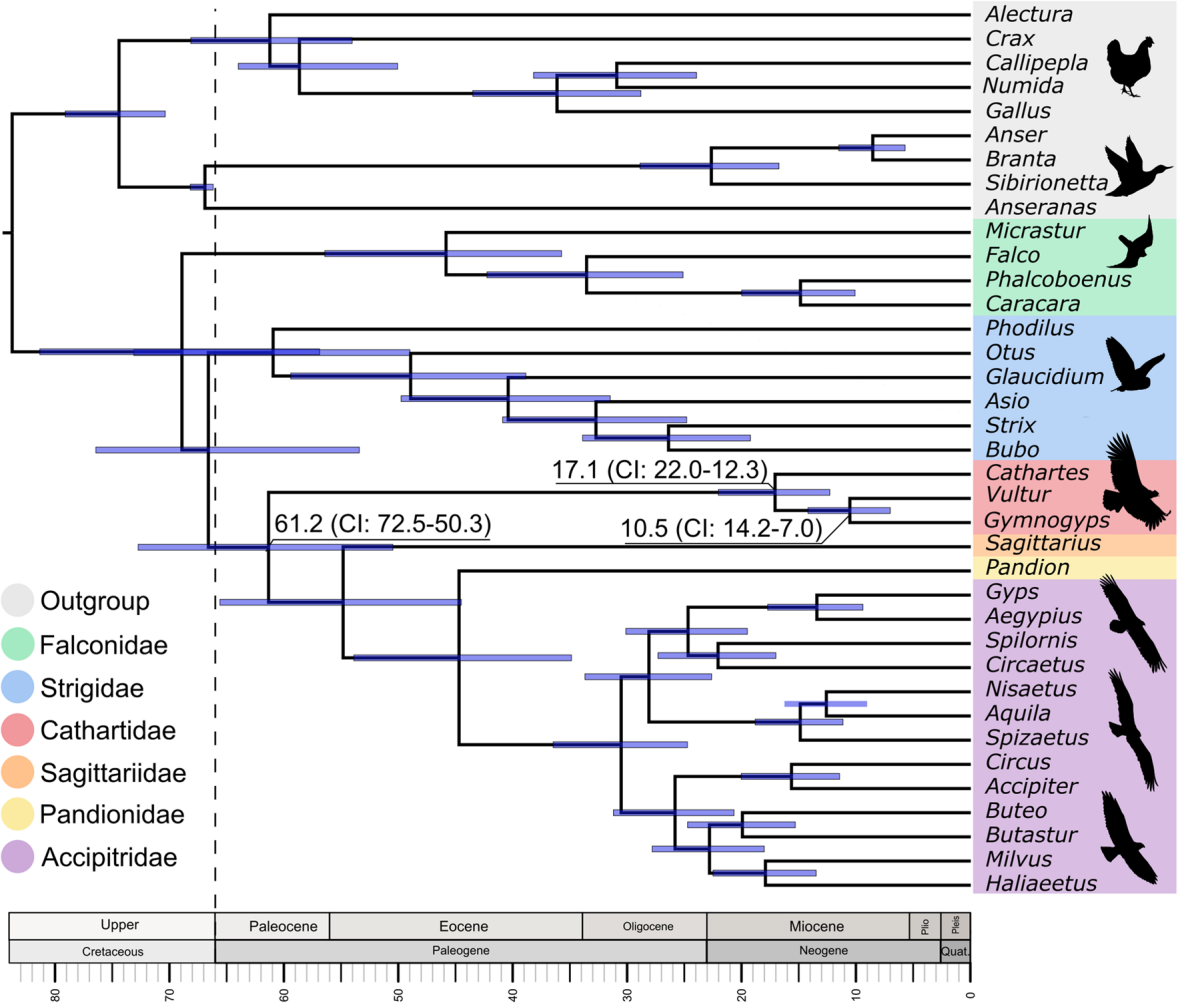
Maximum clade credibility dated tree based on 13 PCGs + 12S and 16S rRNAs (error bars represent 95% CI). Consistent topologies were recovered from ML and BI trees (Figure S5). The inferred ages for Cathartidae are shown. The tree was obtained with FigTree 1.4.4 (https://anaconda.org/bioconda/figtree) and the figure was edited with Inkscape 1.1 (http://www.inkscape.org).

As expected, the first and third domains showed higher intergeneric variability between condors, than the second domain (Fig. 2b). Moreover, the intraspecific variation pattern reported in California condors^18^ (Fig. 2b) is consistent with the expectation that regions exhibiting the greatest interspecific variability tend to vary the most within species^56^. However, we found that most of the intraspecific variability reported in the Andean condor^2,35^ was situated between the Bird Box and CSB1, while the third domain showed an unusually invariant pattern (Fig. 2b), suggesting the existence of functional constraints on this region. Our observation of high similarity between the CR of our NW vultures, with a divergent time as old as ~ 17 My (Fig. 3), along with a similar substitution pattern to those of PCGs, and the observation that STRs could be involved in secondary structures, suggest that the extremely low variability found in the Andean condor could be possibly explained by a slow evolutionary rate, especially in the third domain. Slow rates of evolution of the CR has been previously reported in some Galliforms, Gruiforms, Passeriforms and Charadriiforms^1,56^, while STRs found in the third domain of owls and gulls suggested that these sequences might be functionally essential in terminating mitochondrial genome replication^1,57^. Thus, we suggest that as in the case of the California condor, targeting the first domain of the Andean condor (between ETAS1 and F-BOX), might provide higher intraspecific variability for population genetic studies, especially when using degraded samples for ancient DNA approaches.

### Phylogenetic position and divergence times

Our phylogenetic analyses were consistent across sampling schemes and between ML and BI methods, recovering the same phylogenetic positions for OW and NW vultures, except in the BI analysis of scheme 3 (all PCGs + nonPCGs), that failed to group NW vultures with Accipitriformes (Figure S5). Nodal supports obtained with PCGs (scheme 1) increased with the addition of rRNAs (scheme 2), but decreased with the inclusion of tRNAs (scheme 3), probably due to the conservative nature of these short sequences (Fig. 1a). Thus, we estimated the maximum clade credibility chronogram using 13 PCGs + 2 rRNAs. Overall, our results were similar to recent analysis using whole nuclear genome sequences of raptors^10,11^, splitting our in-group into two basal branches: Afroaves, including Accipitridae (eagles, hawks, kites, harriers, buzzards and OW vultures), Pandionidae (ospreys), Sagittariidae (secretary birds), Cathartidae (NW vultures), Strigidae (owls); and Australaves, including Falconidae (falcons and caracaras; Fig. 3). Hence, the inclusion of mitogenomic data of condors supports the hypothesis that NW and OW vultures belong to Accipitriformes^10,58^ and that vultures are not closely related to Falconiforms as previously believed.

Conspicuous phenotypic differences between NW and OW species, such as the functional hind toe, lack of syrinx, internal separation of the nostrils, or the lack of squirting behavior on the legs in OW vultures, among others^7,9^, suggest that evolutionary constraints (parallel evolution *sensu* neo-Gouldian^59^) are not driving their overall similarities. Moreover, NW vultures seem to be more sensitive to lead contamination than OW species^20^, while the striking tolerance to Diclofenac in the Turkey vulture (> 100 times than in OW vultures) suggests that NW vultures are less vulnerable to the toxic effects of non-steroidal anti-inflammatory drugs^60^. If true, components of genetic variation of the detoxification metabolism have diverged between NW and OW vultures. Future comparative analysis of the nuclear genome between these groups would help to understand to what extent NW and OW vultures exhibit divergent genetic pathways. This information will be critical not only to improve diagnosis and treatments, but also to assist in management plans aimed at maximizing adaptive genetic variability of threatened populations.

Our molecular dating of the origin of NW vultures in the early Paleogene (61.2 Mya; CI 72.5–50.3; Fig. 3) differs from the estimates of recent analysis using whole nuclear genome data of raptor species^11^, but is in agreement with previous studies including wider taxon sampling^9,10,47,58^. A partial explanation for this discrepancy is that Zhou et al.^11^ had a sole representative taxon of NW vultures (Turkey vulture) and lacked genomic resources for the extant representatives of Pandionidae (osprey) and Sagittariidae (secretary bird), resulting in a delayed estimate of the origin of Cathartidae (36 Mya). In fact, the fossil record indicates that Sagittariid, Accipitrid and Pandionid lineages split before the Oligocene, with the oldest putative representatives *Pelargopappus schlosseri, Milvoides kempi* and *Palaeocircus cuvieri*, occurring in the early Oligocene, middle and late Eocene, respectively^9,61,62^. Moreover, our estimated divergence time of NW vultures agrees with the age of the oldest known fossils of the stem group of Cathartidae, *Diatropornis ellioti* and *Paracathartes howardae,* around the early-mid Eocene, almost 40 to 55 Mya, respectively^9,62,63^. Thus, the observed discrepancy between studies highlights the importance of using a combination of proper calibration information and broad taxon sampling^47^. In addition, our determination of the diversification time of extant Cathartids between the mid and late Miocene (22–12 Mya; Fig. 3) is consistent with the earliest record of the condor-like vulture *Hadrogyps aigialeus* from the Barstovian period (~ 16–13 Mya) of California^64^. This result supports the early arising of this group, probably initiated by the paleo-climatic conditions and diversification of megafauna during the Miocene^9,47,65,66^. The historical record shows evidence that NW vultures were highly diverse during that period and harbored large populations, widely distributed until the massive loss of megafauna during the Pleistocene-Holocene transition^64–66^. Andean and California condors persisted into the abrupt ecological changes of the Holocene, possibly due to their foraging flexibility to exploit marine mammals, small terrestrial carcasses, and human-related carrion resources^67^. The oldest undisputed records of extant Andean condors from Pleistocene deposits found in Peru and Chile^68,69^ suggest the recent origin of the species linked to the Andean mountains. Notwithstanding, previous studies proposed that extant condors evolved from a smaller species in North America and expanded to the south crossing the ancient coastline (before the Isthmus of Panama), during the late Miocene—early Pliocene^68,70^ (but see^69^). If true, the distinctive large body size of California and Andean condors may represent a case of rapid convergent evolution driven by the selection pressures of the obligate scavenger lifestyle in mountainous environments. Although more efforts are still needed to elucidate the natural history of vultures, the use of mitogenomic data in conjunction with an increased taxon sampling seems a promising approach to untangle the long‐standing controversial relationships within this group.

### Selective relaxation

**Figure 4:**
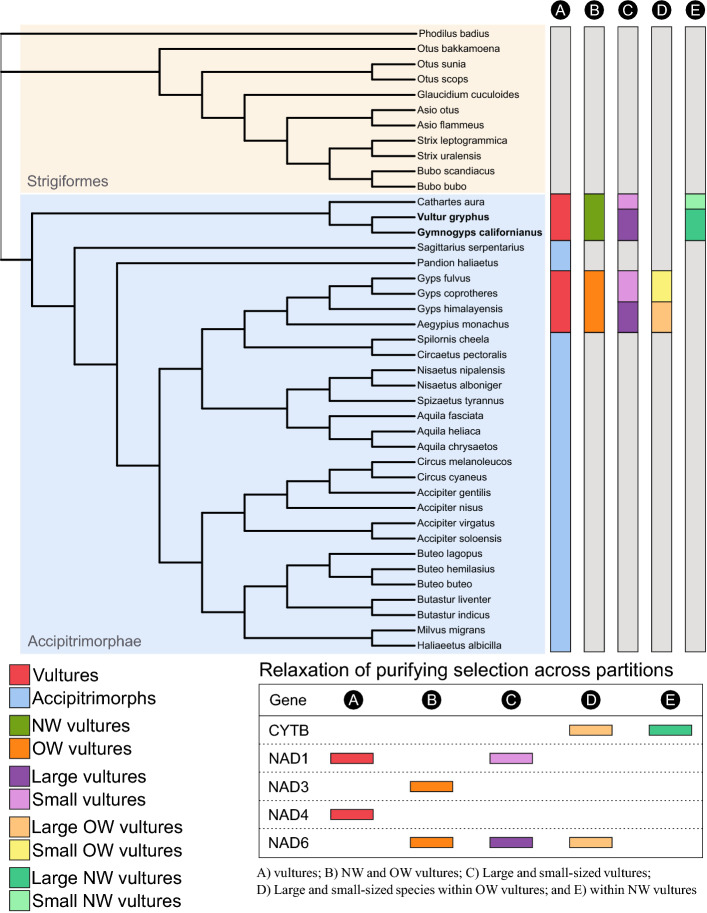
Phylogenetic tree of raptors (Afroaves clade), based on mitochondrial PCGs. Clade partitions (comparisons) are denoted with letters (**A**–**E**), while colored bars indicate target groups of the clade model analysis. The bottom table summarizes the results of genes exhibiting significant selective relaxation for each partition. The tree was obtained with FigTree 1.4.4 (https://anaconda.org/bioconda/figtree) and the figure was edited with Inkscape 1.1 (http://www.inkscape.org).

Our selection analyses suggest that vultures experienced similar levels of genetic constraints to the rest of accipitrimorphs, except for the selective relaxation detected in *ND1* and *ND4* codons in the former group (Fig. 4 and S6; Table S3). A possible explanation for these differences might be related to the contrasting flight abilities of these raptors. Our finding of selective relaxation in those genes is in line with previous studies that found larger substitution rates in *ND* genes in weak versus strong locomotive birds due to the stronger selective pressure to eliminate deleterious mutations in the latter^71^. Thus, it is possible that the stronger purifying selection observed in non-vulture accipitrids is related to the active metabolism of flapping flight, while the specialization of soaring flight in vultures resulted in the relaxation of selective constraints on the energy metabolism^23,24^. In fact, recent reports demonstrated that vultures are able to fly with a strikingly minimal amount of flapping^72^, which in the Andean condor can be as low as 1% of the total flight time, a record for any living flying bird^25^. In addition to the locomotive strategy, our results also suggested the implication of phylogenetic constraints influencing mitochondrial evolution. NW vultures showed unique and radical non-synonymous substitutions in several genes compared to the rest of Accipitrimorphs (Figure S7). Furthermore, our ω estimates for NW species were smaller, suggesting stronger purifying selection (Figure S6), while our CmC analysis showed that OW vultures exhibited significant relaxation in *ND3* and *ND6* codons (Fig. 4; Table S3). This is consistent with our observation of large numbers of non-synonymous substitutions fixed in these genes of OW vultures (Figure S7), and lower ω values in the terminal branches of NW species compared to OW vultures (0.04 and 0.28, respectively; *P* < 0.005 in both cases). Notwithstanding, a larger number of species is needed to confirm the phylogenetic inertia of substitution patterns.

Our CmC analysis of selective signals according to the body size of vultures revealed that larger species exhibited significant relaxation in *ND6* codons, while smaller species showed selective relaxation in *ND1* codons (Fig. 4). However, our regression analyses showed no relation between the body mass of vultures and their ω values in any gene (*P* > 0.22 in all cases). Furthermore, after dis-aggregating the CmC analysis within OW and NW vultures, our results suggested that the observed relaxation pattern might be explained by the strong relaxation signal of large OW species, especially in *ND6* (Fig. 4, Table S3). This was further confirmed by our BM and BSM analysis (*P *< 0.001), suggesting the involvement of positive selection acting on *ND6* sites (Table S3). Thus, our results propose that the evolution of body size in this guild is not associated with a common or simple mitochondrial genetic basis. Given that large OW species inhabit higher mountains and fly at higher elevations than condors, our detection of positively selected *ND6* sites suggests a possible signal of mitochondrial adaptation to hypoxia. Previous studies have shown that positive selection of the NADH dehydrogenase complex plays an important role in adaptation to high-altitude in mammals and in galliform and passeriform birds^6,73^. If true, our observed selection footprint should be also present in smaller high-altitude vultures. Thus, the inclusion of the mitogenome of the Ruppell’s griffon (*Gyps rueppelli*) will be instrumental to test this hypothesis, as this medium-sized vulture (~ 7 kg) can fly as high as 11.300 masl and exhibit specialized haemoglobins with a higher oxygen affinity^4,31^. Finally, our CMC and BM analyses focused on NW vultures revealed a selective relaxation signal in condor´s *CYTB* gene, respect the Turkey vulture´s ortholog (*P* = 0.028; Table S3). This result could be related to the migratory behaviour of Turkey vultures^74^, which might exert higher selective pressures on mt-genes related to the energy metabolism of this small vulture.

Overall, our results suggest that the exceptional specialization of soaring flight of vultures has compensated the evolution of body mass without pressing directional changes on mtDNA. The energetic cost of flapping flight is expected to be extremely high compared to soaring or gliding flight, and lighter species tend to flap more than large ones^25^. Given that vulture species are among the largest flying birds and evolved the most extreme use of soaring flight^23–25,74^, the relaxation of selective constraints on mt-genes may indicate the degeneration of flapping flight ability. Our results are consistent with previous studies that found small differences in the energy consumption between flight and resting in large vultures^25,72^, suggesting that low metabolic rates might have buffered the selection pressure on mitochondrial efficiency. Notwithstanding, nuclear genomic sequences and an increased number of species are needed to better understand how the evolution of the obligate scavenger lifestyle affected metabolic selection.

## Supplementary Information

Supplementary Information.

## Data Availability

Details of data analyses and accession numbers for sequences retrieved from GenBank have been uploaded as part of the Supplementary Material. Additionally, mitogenomic sequences generated in this study have been deposited in the GenBank database under accession No. MZ223429 and BK059163.
