# Supplementary material for: Mitogenomic analysis of extant condor species provides insight into the molecular evolution of vultures
Source: Sci Rep. 2021 Aug 24;11:17109. doi: 10.1038/s41598-021-96080-6 (PMC8384887; doi:10.1038/s41598-021-96080-6)
Supplement: Supplementary file 1 — Supplementary Information. [file 41598_2021_96080_MOESM1_ESM.docx]

Supplementary Material

Title Mitogenomic analysis of extant condor species provides insight into the molecular evolution of vultures

Journal *Scientific Reports*

Authors D. De Panis*^1,2^, S. A. Lambertucci^1^, G. Wiemeyer^1,3,4,5^, H. Dopazo^2^, F. C. Almeida^2^, C. Mazzoni^6^, M. Gut^7^, I. Gut^7^, J. Padró*^1^

Affiliations ^1^Grupo de Investigaciones en Biología de la Conservación, INIBIOMA, Universidad Nacional del Comahue -CONICET, Bariloche, 8400, Argentina

^2^ Instituto de Ecología, Genética y Evolución de Buenos Aires, Consejo Nacional de Investigaciones Científicas y Técnicas, Ciudad Autónoma de Buenos Aires, Argentina.

^3^ Ecoparque Buenos Aires-Argentina, República de la India 3000, CABA, CP,1425, Ciudad Autónoma de Buenos Aires, Argentina.

^4^ Present Address: Universidad de Buenos Aires, Facultad de Ciencias Veterinarias, Hospital Escuela. Av. Chorroarín 280, CP 1427, Ciudad Autónoma de Buenos Aires, Argentina.

^5^ Fundación Cabure-í., Mcal Antonio Sucre 2842, CP 1428, Ciudad Autónoma de Buenos Aires, Argentina.

^6^ Berlin Center for Genomics in Biodiversity Research, Berlin, Germany.

^7^ CNAG‐CRG, Centre for Genomic Regulation, Barcelona Institute of Science and Technology, Spain

Corresponding Authors: *Diego De Panis,* [*dndepanis@ege.fcen.uba.ar*](mailto:dndepanis@ege.fcen.uba.ar) *Julian Padró,* [*padrojulian@comahue-conicet.gob.ar*](mailto:padrojulian@comahue-conicet.gob.ar)

**Extended Methods**

**Mitochondrial genome assembly**

We mapped the sequences of both the California and Andean condor against the reference mitogenome of *C. aura* and *G. gallus* (Table S1), using Bowtie2 2.3.5.1 (Langmead & Salzberg 2012) and Minimap2 2.17 (Li 2018) for short and long reads, respectively. Our assemblies obtained with NOVOPlasty v4.2 (Dierckxsens et al. 2016), GetOrganelle v1.7.1 (Jin et al. 2020) and UniCycler v0.4.8 (for the Andean condor; Wick et al. 2017) were aligned to *C. aura* and *G. gallus* mitochondrial reference genomes using Mauve v2.4.0 (Darling et al., 2004) and MAFFT v7.3 (Katoh & Standley 2013) to analyze and build a coherent consensus sequences. After the proper ordering and orientation of the sequences, the difference between Andean condor assemblies was found in the length of a repetitive segment of the control region. Since both GetOrganelle and UniCycler algorithms were reported as circulars, we selected the first approach as the most conservative option because it also used a partial sequence of the control region as seed. A further inspection after mapping our raw-reads showed similar consistency between the two alternatives. For the California condor assemblies, GetOrganelle was reported as circular and NOVOPlasty was used to check consistency. Since Hendrickson et al. (2003) reported a conserved order in a partial mitochondrial sequence between Andean condor and chicken, we used *G. gallus* mitochondrion in addition to *C. aura* to check annotation consistency. All genes (tRNAs, rRNAs and PCGs), as well as the control region (CR) were manually curated and genomic assembly was schematized with OGDRAW 1.3.1 (Greiner et al. 2019).

**Selection Analysis**

We assessed selective footprints in the mitochondrial PCGs of vultures in a phylogenetic context of the Afroaves clade by estimating the nonsynonymous-to-synonymous substitution rate ratio (ω=dN/dS). For this, we built a phylogenetic tree including all available species in the order Strigiformes (Owls), Cathartiformes and Accipitriformes (the last two conforming the clade Accipitrimorphae that encompass vultures, eagles and hawks, among other raptors). We used the PAML wrapper EasyCodeML 1.31 (Gao et al. 2019) using the codon-aware filtered gene alignments to fit substitution models to the data for ω estimation. Because mitochondrial PCGs tend to be highly conservative, we used likelihood ratio tests (LRTs) to compare the fit of Clade model C (CmC) to detect subtle differences in site-specific selective constraint among clade partitions, with M2a_rel as the null-model. In the CmC model, codons can experience either purifying (0<ω<1) or neutral (ω=1) selection across the entire phylogeny, or unconstrained / positive selection (ω>0) in the pre-selected partitions, while in the M2a_rel model, codons can adopt the same three classes of selective pressures equally across the entire phylogeny. Thus, we used the PAML algorithm to make several partition comparisons. First, to rule out phylogenetic constraints we compared vultures and the rest of the Accipitrimorphae as two 'foreground' branches partitions, with Strigiformes as 'background' branch (A), and on the other hand, New and Old World vultures as two foreground partitions, with the rest of the phylogeny as 'background' (B). We also analyzed selective constraints across vultures based on the species body size, combining all large and small species, and within each family (see Figure 4). First, we set as two 'foreground' partitions the branches of large-sized (*V. gryphus, G. californianus, G. himalayensis and A. monachus*) and small species (*C. aura, G. fulvus and G. coprotheres*), respectively, using the rest of the tree as 'background' (C). For the Old World vultures, we assigned the branches of *G. himalayensis* and *A. monachus* as large-sized and *G. fulvus* and *G. coprotheres* as small vultures (D). Similarly, for New World vultures, we selected as two 'foreground' partitions the branches of *V. gryphus* and *G. californianus* as large species, and *C. aura* as small one, with the rest of the phylogeny as 'background' (E). In addition, for every gene resulting in LRT p-value < 0.1, we tested the fitting of equivalent Branch models and Branch-Site models (for one species at a time). For the Branch model, we compared the fitting of the Two-ratio model (Model 2), which estimates a single ω value for each specified branch, versus Model 0 (same ω value for all branches). For the Branch-Site model, we compared the fitting of a null-model that restricts diversifying selection, with Model A. The latter estimates ω allowing codons to adopt purifying or neutral selection across the phylogeny, and codons that show purifying or neutral selection on the 'background' partitions, but positive selection on the selected branch.

Tables

Table S1. Mitogenome accession number of bird species investigated in this work

| **Family** | **Species name** | **Accession** |  | **Family** | **Species name** | **Accession** |
| --- | --- | --- | --- | --- | --- | --- |
| Megapodiidae | Alectura lathami* | NC_007227.1 |  | Cathartidae | Gymnogyps californianus* | BK059163 |
| Odontophoridae | Callipepla squamata* | NC_029340.1 |  | Cathartidae | Cathartes aura* | NC_007628.1 |
| Cracidae | Crax rubra* | NC_024618.1 |  | Cathartidae | Vultur gryphus* | MZ223429 |
| Phasianidae | Gallus gallus spadiceus* | NC_040902.1 |  | Pandionidae | Pandion haliaetus* | NC_008550.1 |
| Numididae | Numida meleagris* | NC_034374.1 |  | Sagittariidae | Sagittarius serpentarius* | NC_023788.1 |
| Anatidae | Sibirionetta formosa* | NC_015482.1 |  | Accipitridae | Gyps coprotheres | MF683387.1 |
| Anatidae | Anser cygnoides* | NC_023832.1 |  | Accipitridae | Gyps himalayensis | NC_039095.1 |
| Anseranatidae | Anseranas semipalmata* | NC_005933.1 |  | Accipitridae | Aegypius monachus* | KF682364.1 |
| Anatidae | Branta canadensis* | NC_007011.1 |  | Accipitridae | Gyps fulvus* | NC_036050.1 |
| Falconidae | Phalcoboenus australis* | NC_031897.1 |  | Accipitridae | Circus cyaneus | KX925606.1 |
| Falconidae | Caracara plancus* | NC_044672.1 |  | Accipitridae | Nisaetus nipalensis | NC_007598.1 |
| Falconidae | Falco peregrinus* | NC_000878.1 |  | Accipitridae | Accipiter gentilis* | NC_011818.1 |
| Falconidae | Micrastur gilvicollis* | NC_008548.1 |  | Accipitridae | Aquila heliaca* | NC_035806.1 |
| Strigidae | Asio otus | NC_039736.1 |  | Accipitridae | Buteo buteo* | NC_003128.3 |
| Strigidae | Bubo scandiacus | NC_038220.1 |  | Accipitridae | Butastur indicus* | NC_032362.1 |
| Strigidae | Otus scops | NC_028162.1 |  | Accipitridae | Circus melanoleucos* | NC_035801.1 |
| Strigidae | Otus sunia | NC_041422.1 |  | Accipitridae | Circaetus pectoralis* | NC_052805.1 |
| Strigidae | Strix leptogrammica | KC953095.1 |  | Accipitridae | Haliaeetus albicilla* | NC_040858.1 |
| Strigidae | Asio flammeus* | NC_027606.1 |  | Accipitridae | Milvus migrans* | NC_038195.1 |
| Strigidae | Bubo bubo* | NC_038219.1 |  | Accipitridae | Nisaetus alboniger* | NC_007599.1 |
| Strigidae | Glaucidium cuculoides* | NC_034296.1 |  | Accipitridae | Spilornis cheela* | NC_015887.1 |
| Strigidae | Otus bakkamoena* | NC_028163.1 |  | Accipitridae | Spizaetus tyrannus* | NC_052803.1 |
| Tytonidae | Phodilus badius* | NC_023787.1 |  | Accipitridae | Accipiter nisus | NC_025580.1 |
| Strigidae | Strix uralensis* | NC_038218.1 |  | Accipitridae | Accipiter soloensis | KJ680303.1 |
|  |  |  |  | Accipitridae | Accipiter virgatus | NC_026082.1 |
|  |  |  |  | Accipitridae | Aquila chrysaetos | NC_024087.1 |
|  |  |  |  | Accipitridae | Aquila fasciata | NC_029188.1 |
|  |  |  |  | Accipitridae | Buteo hemilasius | NC_029377.1 |
|  |  |  |  | Accipitridae | Buteo lagopus | NC_029189.1 |
|  |  |  |  | Accipitridae | Butastur liventer | NC_032363.1 |

(* Representative species of their genus used for phylogenetic reconstructions).

Table S2. Positions (referenced to D-loop of *V.gryphus;* Fig.2b) and sequences of conserved elements of the control region of six vulture species

Species POLY-C start 25bp ETAS-1 start 68bp FBOX start 386bp

*V. gryphus* **AC**CCCCCCC**TA**CCCCCCC**ATA** TA**C**TGT**G**CATTA**ATA**TAT**T**T**A**CCCCATA G**G**TCTTC**TCA**CG**A**GAAATCAGCAACC**C**

*G. californianus* **GA**CCCCCCC**TA**CCCCCCC**ATG** TA**T**TGT**A**CATTA**ATA**TAT**T**T**A**CCCCATA G**T**TCTTC**TCA**CG**T**GAAATCAGCAACC**C**

*C. aura*  **TA**CCCCCCC**TA**CCCCCCC**ATA** TA**C**TGT**G**CATTA**ATA**TAT**T**T**A**CCCCATA G**T**TCTTC**TCA**CG**T**GAAATCAGCAACC**C**

*G. fulvus* **CC**CCCCCCC**TA**CCCCCCC**C - -** CA**T**TGT**A**CATTA**CAC**TAT**T**T**G**CCCCATA G**G**TCTTC**CTC**CG**A**GAAATCAGCAACC**G**

*G. himalayensis* **TC**CCCCCCC**CC**CCCCCCC**CCC** CA**T**TGT**A**CATTA**CAC**TAT**C**T**G**CCCCATA G**G**TCTTC**CTC**CG**A**GAAATCAGCAACC**G**

*G. coprotheres* **CC**CCCCCCC**CC**CCCCCCC**CAT** CA**T**TGT**A**CATTA**CAC**TAT**T**T**G**CCCCATA G**G**TCTTC**CTC**CG**A**GAAATCAGCAACC**G**

EBOX start 430bp DBOX start 492bp CBOX start 538bp

**ATATG**AC**T**AGCTTCAGGA**C**C**A** CCTCTGGTTCCT**C**T**G**TCA**G**GGCCA**TTC** TTG**C**TCTT**C**AC**AG**A**A**ACATCTGGTA**- - - -**

**TCACG**AC**C**AGCTTCAGGA**T**C**A** CCTCTGGTTCCT**A**T**T**TCA**G**GGCCA**TTC** TTG**T**TCTT**C**AC**AG**A**A**ACATCTGGTA**- - - -**

**ACACG**AC**C**AGCTTCAGGA**C**C**A** CCTCTGGTTCCT**A**T**A**TCA**G**GGCCA**TTC** TTG**C**TCTT**C**AC**AG**A**A**ACATCTGGTA**- - - -**

**ACGTT**AC**T**AGCTTCAGGA**T**C**T** CCTCTGGTTCCT**A**T**A**TCA**A**GGCCA**CAA** TTG**C**TCTT**T**AC**GA**A**T**ACATCTGGTATGGC

**ACGTT**AC**T**AGCTTCAGGA**T**C**T** CCTCTGGTTCCT**A**T**A**TCA**A**GGCCA**CAA** TTG**C**TCTT**T**AC**GA**A**T**ACATCTGGTATGGC

**ACGTT**AC**T**AGCTTCAGGA**T**C**T** CCTCTGGTTCCT**A**T**A**TCA**A**GGCCA**CAA** TTG**C**TCTT**T**AC**GA**A**T**ACATCTGGTATGGC

CSBa start 619bp CSBb start 726bp Bird Box start 775bp CSB1 start 836bp

TGGTTCCC**- -**TTTTTTTTTGGGG**C G**CAAGA**A**T**CCC**T**G**AATGA**G**ACGGTT**T** CACTGATGCACT**T**TGC TA**T**TTAG**T**GAA**T**GCTTG**TT**GGACATG**A**

TGGTTCCC**- -**TTTTTTTTTGGGG**C T**CAAGA**A**T**TAC**T**G**AATGA**G**ACGGTT**T** CACTGATGCACT**T**TGC TA**T**TTAG**T**GAA**T**GCTTG**TT**GGACATG**A**

TGGTTCCC**- -**TTTTTTTTTGGGG**C T**CAAGA**A**T**TAC**T**G**AATGA**G**ACGGTT**T** CACTGATGCACT**T -**GC TA**T**TTAG**T**GAA**T**GCTTG**TT**GGACATG**T**

TGGTTCCCTTTTTTTTTTTGGGG**A T**CAAGA**G**T**TGA**T**T**AATGA**A**ACGGTT**G** CACTGATGCACT**G**T **-**C TA**T**TTAG**G**GAA**G**GCTTG**GG**GGACATG**T**

TGGTTCCCTTTTTTTTTTTGGGG**T T**CAAGA**G**T**TGA**T**T**AATGA**G**ACGGTT**G** CACTGATGCACT**G**T **-**C TA**A**TTAG**T**GAA**T**GCTTG**TG**GGACATG**T**

TGGTTCCC**-**TTTTTTTTTTGGGG**T T**CAAGA**G**T**TGA**T**T**AATGA**G**ACGGTT**G** CACTGATGCACT**G**T **-**C TA**T**TTAG**T**GAA**T**GCTTG**TG**GGACATG**T**

Table S3**.** Summary of results of Clade Model C (CmC: *P* < 0.1), Branch (BM) and Branch-Site Model (BSM) analyses (*P* < 0.05).

| CmC Partition A: f1=Vultures, f2=Other accipitrimorphs | | | | | |  |  | CmC Partition D: f1=Large OW, f2=Small OW | | | | | |  |  | BM Partition B: 1=NW, 2=OW | | | | |
| --- | --- | --- | --- | --- | --- | --- | --- | --- | --- | --- | --- | --- | --- | --- | --- | --- | --- | --- | --- | --- |
| Gene | Site class 2 | | | | LRT P-value |  |  | Gene | Site class 2 | | | | LRT P-value |  |  | Gene | Estimates of parameters | | | LRT P-value |
|  | ωb | ω2f1 | ω2f2 | prop. |  |  |  |  | ωb | ω2f1 | ω2f2 | prop. |  |  |  |  | ω0 | ω1 | ω2 |  |
| ATP8 | 0.1350 | 0.2620 | 0.2523 | 0.4476 | 0.0515 |  |  | ATP6 | 0.1556 | 0.4378 | 0.2750 | 0.1741 | 0.0992 |  |  | **NAD6** | 0.0557 | 0.0401 | 0.2862 | **<0.0001** |
| **NAD1** | 0.1297 | 0.1483 | 0.0811 | 0.1985 | **0.0056** |  |  | ATP8 | 0.2121 | 0.0000 | 1.3165 | 0.3952 | 0.0646 |  |  |  |  |  |  |  |
| **NAD4** | 0.1317 | 0.1261 | 0.0979 | 0.2688 | **0.0246** |  |  | **CYTB** | 0.0651 | 0.2331 | 0.0935 | 0.2577 | **0.0027** |  |  | BM Partition C: 1=Large vultures, 2=Small vultures | | | | |
| NAD4L | 0.1095 | 0.1215 | 0.1429 | 0.3106 | 0.5050 |  |  | **NAD6** | 0.1822 | 4.6300 | 0.3021 | 0.2365 | **0.0000** |  |  | Gene | Estimates of parameters | | | LRT P-value |
|  |  |  |  |  |  |  |  |  |  |  |  |  |  |  |  |  | ω0 | ω1 | ω2 |  |
| CmC Partition B: f1=NW vultures, f2=OW vultures | | | | | |  |  | CmC Partition E: f1=Large NW, f2=Small NW | | | | | |  |  | **NAD1** | 0.0249 | 0.0362 | 0.0863 | **0.0313** |
| Gene | Site class 2 | | | | LRT P-value |  |  | Gene | Site class 2 | | | | LRT P-value |  |  | **NAD6** | 0.0558 | 0.1422 | 0.0384 | **0.0025** |
|  | ωb | ω2f1 | ω2f2 | prop. |  |  |  |  | ωb | ω2f1 | ω2f2 | prop. |  |  |  |  |  |  |  |  |
| ATP6 | 0.1603 | 0.0904 | 0.2711 | 0.1742 | 0.0776 |  |  | **CYTB** | 0.0734 | 0.0318 | 0.0284 | 0.2496 | **0.0340** |  |  | BM Partition D: 1=Large OW, 2=Small OW | | | | |
| **NAD3** | 0.1184 | 0.0452 | 0.1942 | 0.2877 | **0.0344** |  |  |  |  |  |  |  |  |  |  | Gene | Estimates of parameters | | | LRT P-value |
| **NAD6** | 0.1909 | 0.1295 | 0.8280 | 0.2498 | **0.0023** |  |  | BSM : A. monachus | | | | | |  |  |  | ω0 | ω1 | ω2 |  |
|  |  |  |  |  |  |  |  | Gene | Site class ω1 | | | | LRT P-value |  |  | **CYTB** | 0.019 | 0.068 | 0.025 | **0.0004** |
| CmC Partition C: f1=Large vultures, f2=Small vultures | | | | | |  |  |  | 2a | 2b | add. prop. | pos. sites |  |  |  | **NAD6** | 0.055 | 0.365 | 0.069 | **0.0000** |
| Gene | Site class 2 | | | | LRT P-value |  |  | CYTB | 999 | 999 | 0.0153 | 315 | 0.0238 |  |  |  |  |  |  |  |
|  | ωb | ω2f1 | ω2f2 | prop. |  |  |  | NAD6 | 999 | 999 | 0.0768 | 2,3,5,9,12 | <0.0001 |  |  |  |  |  |  |  |
| ATP6 | 0.1588 | 0.3012 | 0.0773 | 0.1738 | 0.0656 |  |  |  |  |  |  |  |  |  |  |  |  |  |  |  |
| NAD1 | 0.1004 | 0.1467 | 0.3472 | 0.1984 | 0.0631 |  |  | BSM : G. himalayensis | | | | | |  |  |  |  |  |  |  |
| **NAD6** | 0.1731 | 0.5483 | 0.0606 | 0.2413 | **0.0103** |  |  | Gene | Site class ω1 | | | | LRT P-value |  |  |  |  |  |  |  |
|  |  |  |  |  |  |  |  |  | 2a | 2b | add. prop. | pos. sites |  |  |  |  |  |  |  |  |
|  |  |  |  |  |  |  |  | NAD6 | 999 | 999 | 0.0499 | 4,5,9,11,12 | <0.0001 |  |  |  |  |  |  |  |

Figures


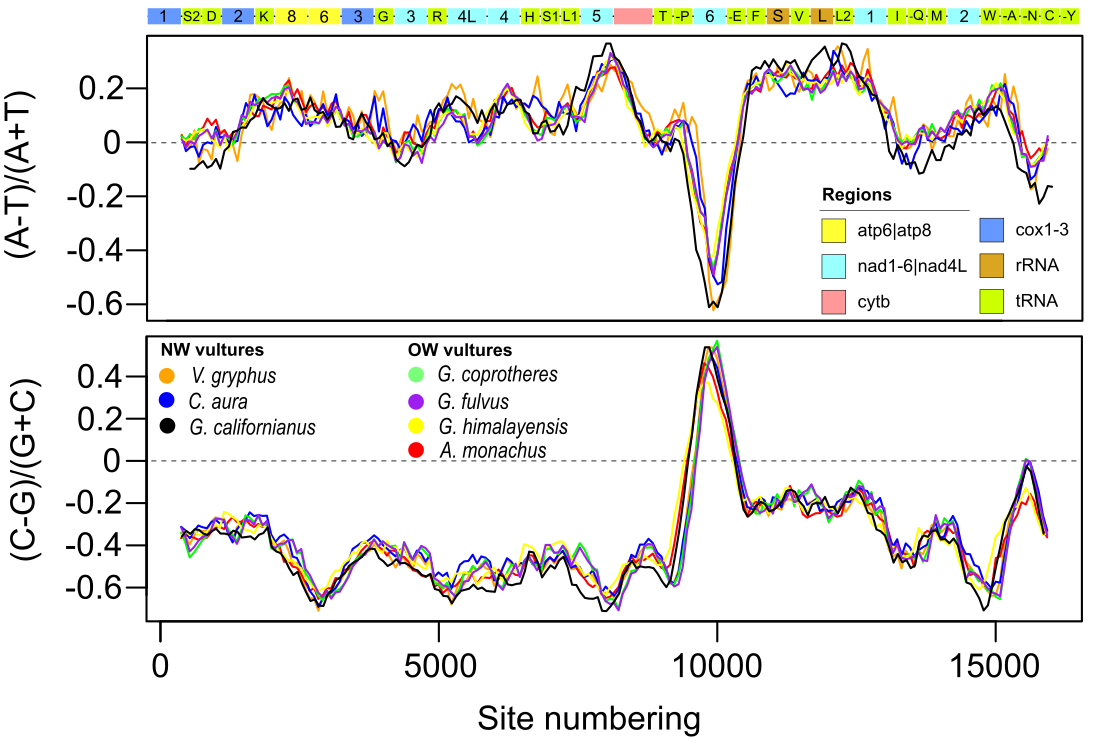


Figure S1. Nucleotide skews of the mitogenomes of New and Old world vulture species. Pearson correlation analysis between species was significant in all cases (r > 0.9 and *P* < 0.001).


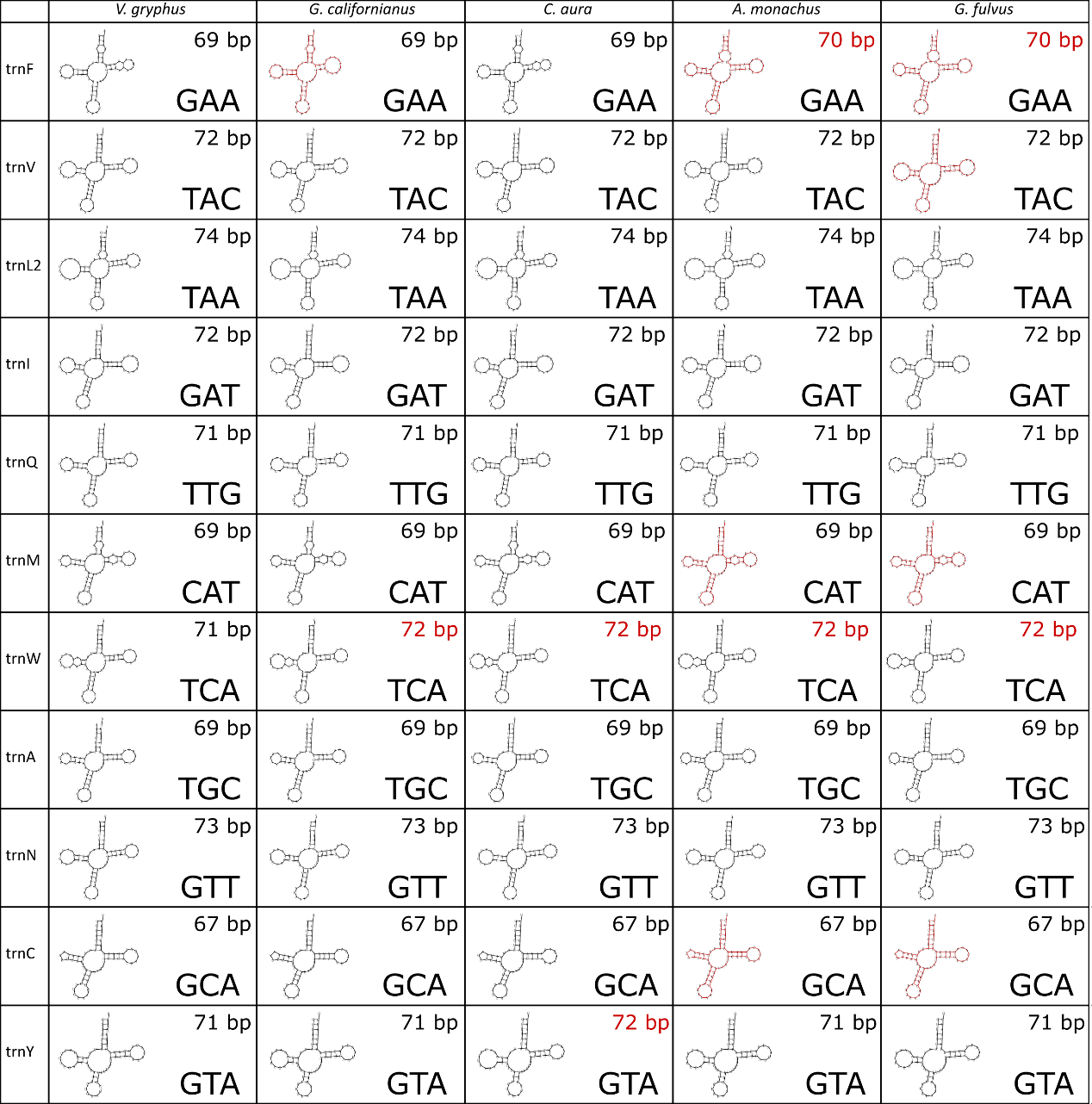


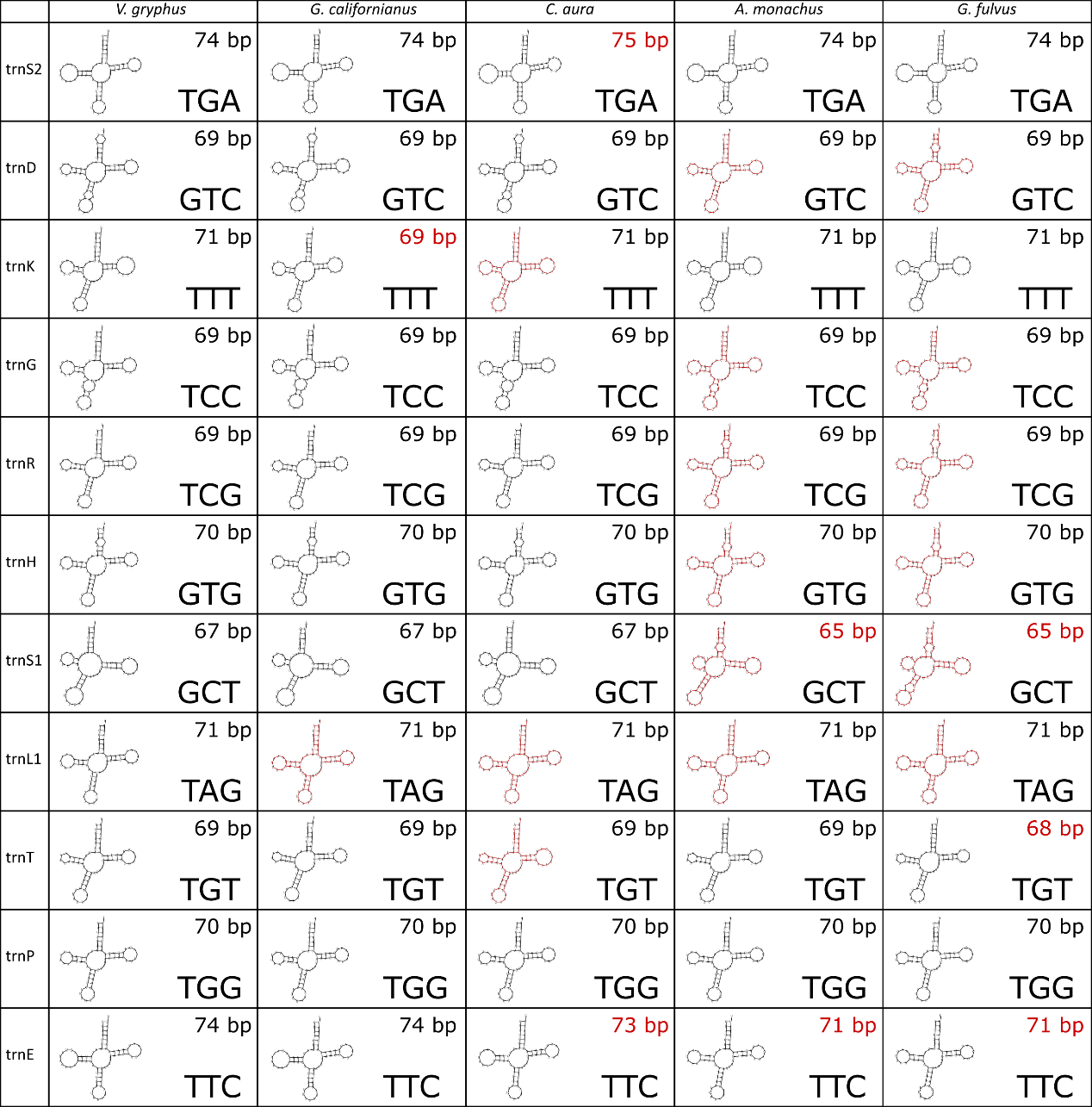


Figure S2. Comparison of predicted secondary structure of tRNAs for five genera of vultures. Differences in length and structure respect to *V. gryphus* are highlighted in red.


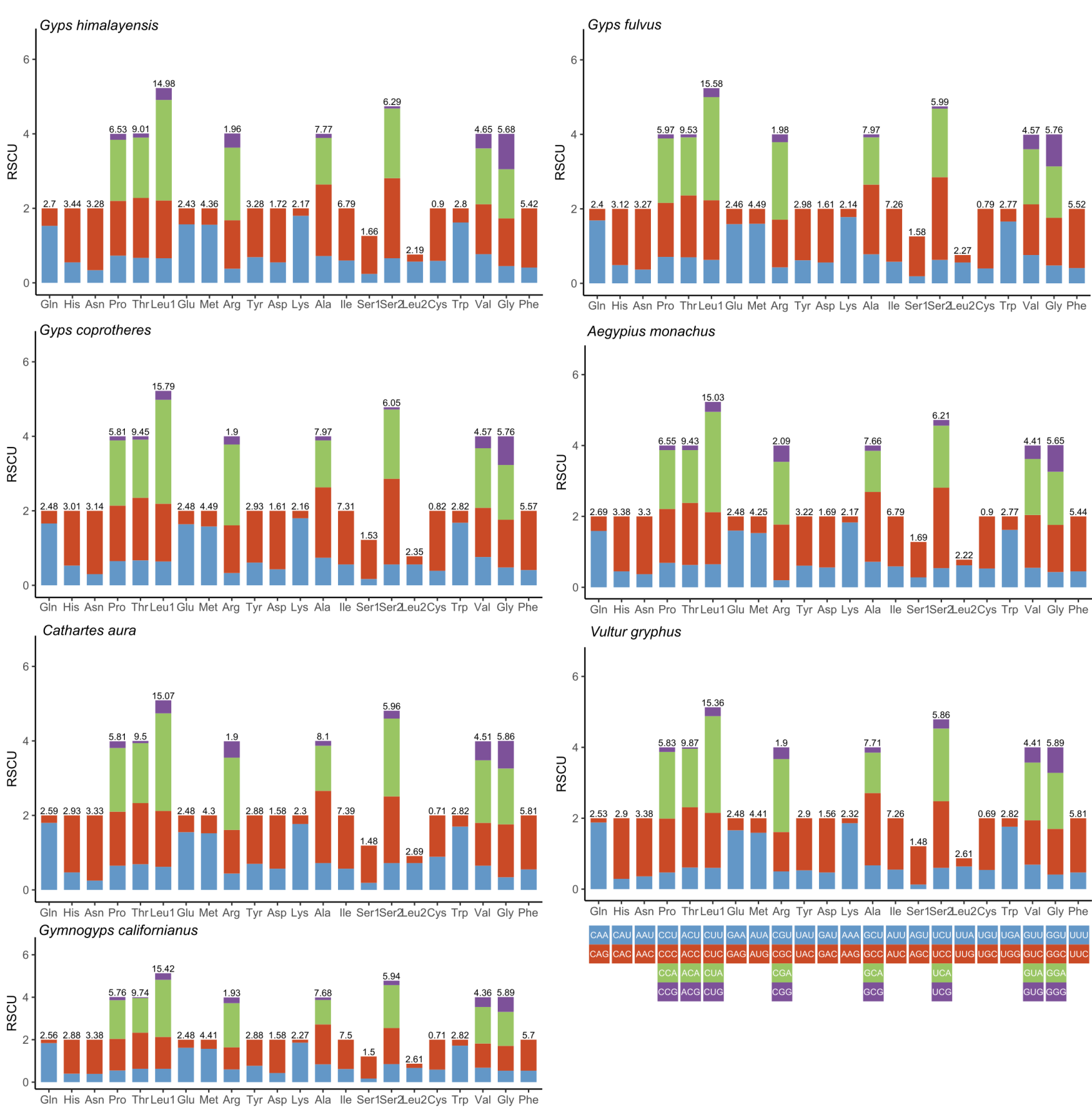


Figure S3. Relative synonymous codon usage for 7 vulture species. Codon families are colored and amino acid usage are denoted on top of the bars.


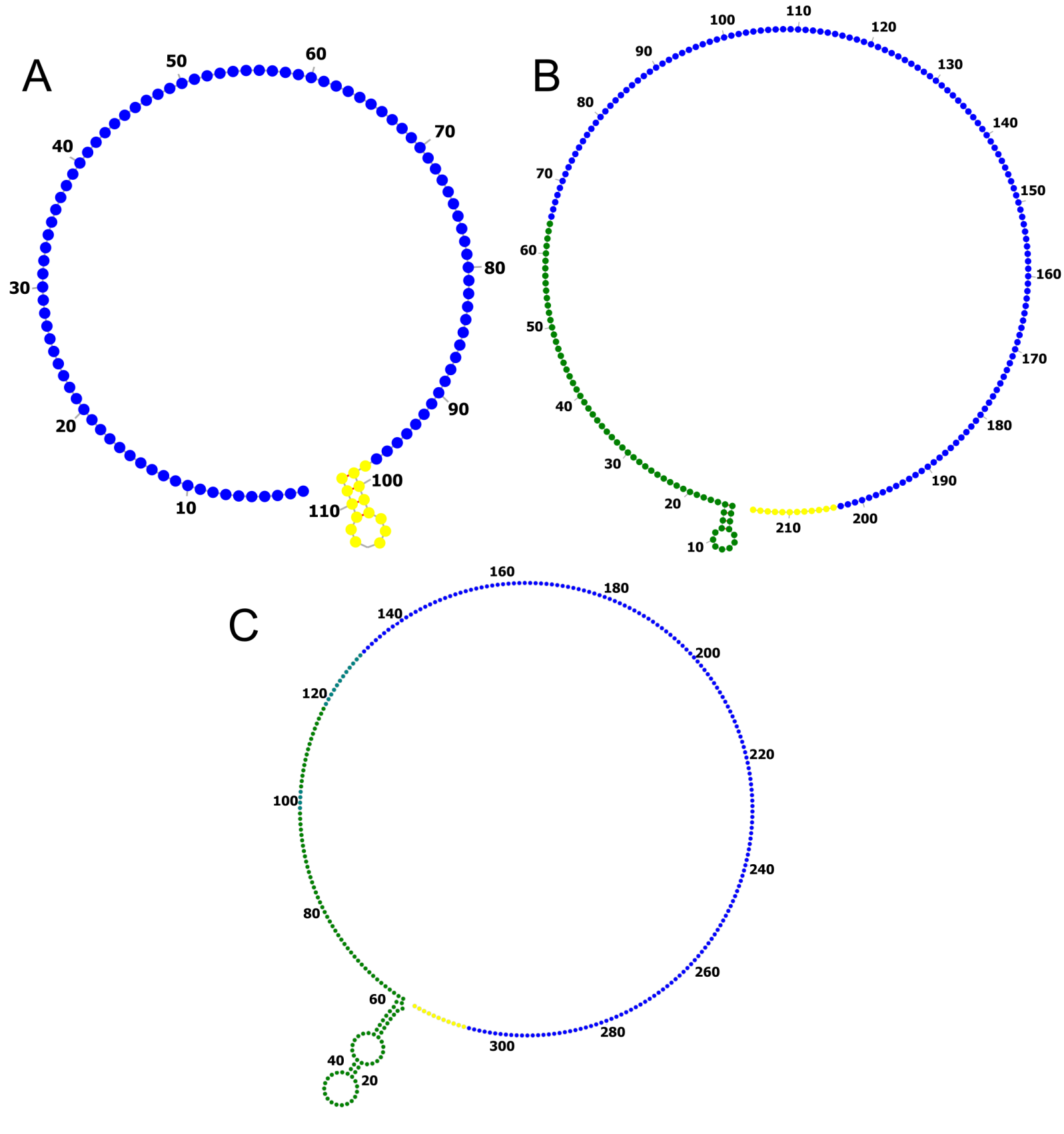


Figure S4. Secondary structure of the third domain of the control region involving the STRs in the turkey vulture (A) and Andean condor (B) and California condor (C). Blue color denotes the STRs region, while yellow indicate the 3’ end region. The stem structure in the California and Andean condors was found in the terminal region of the third domain adjacent to the STRs (green).


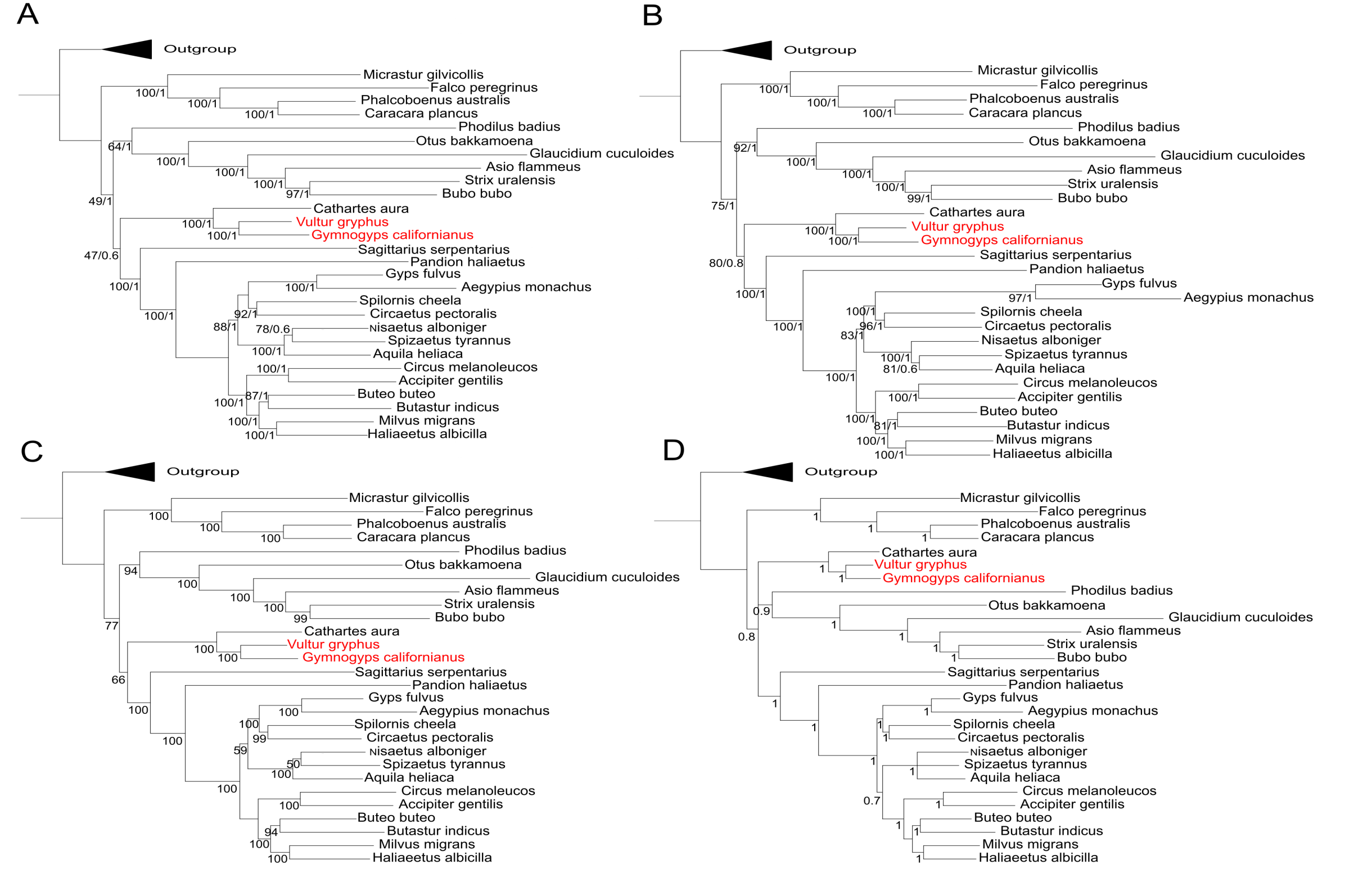
Figure S5. Results of phylogenetic trees based on maximum likelihood (ML) and Bayesian inference (BI) analyses for 28 genera of raptors. Tree topologies of ML and BI were identical in PCGs (A) and PCGs+Rrnas (B), but differed in PCGs+non PCGs (C: ML; D: BI). Bootstrap support values from ML and Bayesian posterior probability are shown on the nodes.


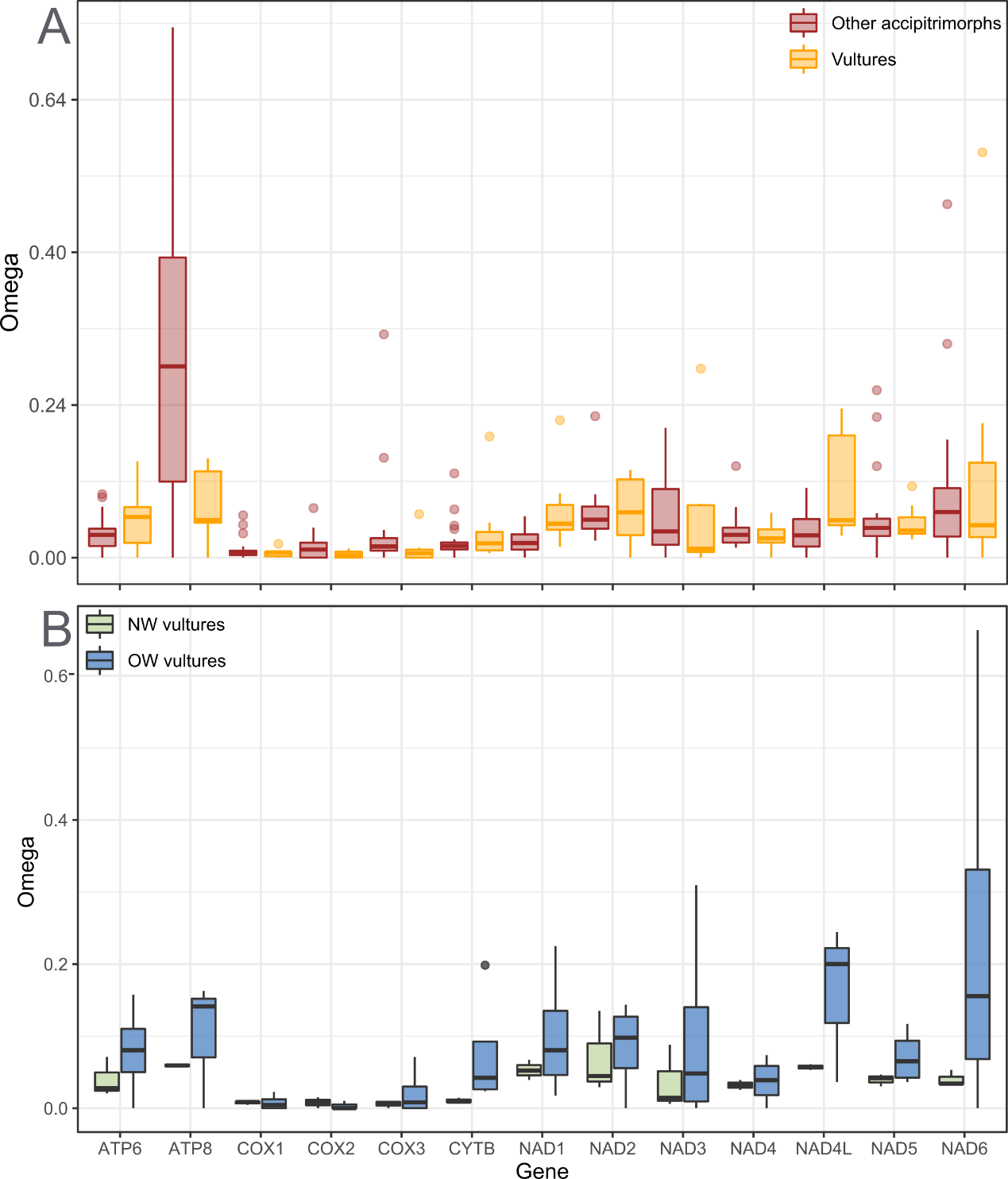
Figure S6. Comparisons of terminal-branch ω-values estimated with free-ratios Model 1 between vultures and the rest of accipitrimorph species (A) and between NW and OW vultures (B).


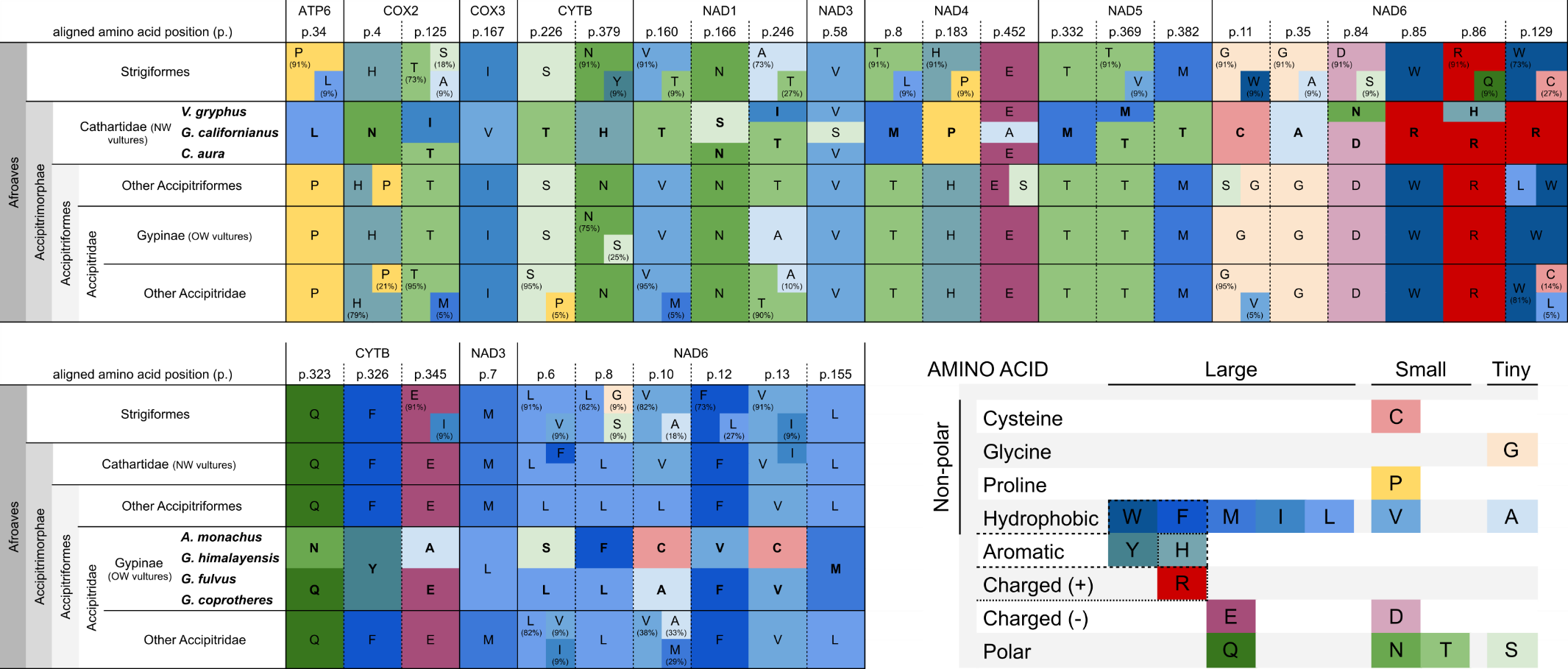


Figure S7. Amino acid substitution pattern across analyzed species of raptors. For NW vultures the analysis was conducted across all PCGs, while in the OW vultures the analysis was restricted to genes showing significant selective relaxation.

**References**

Darling, A. C., Mau, B., Blattner, F. R., & Perna, N. T. (2004). Mauve: multiple alignment of conserved genomic sequence with rearrangements. Genome research, 14(7), 1394-1403.

Dierckxsens, N., Mardulyn, P., & Smits, G. (2017). NOVOPlasty: de novo assembly of organelle genomes from whole genome data. Nucleic acids research, 45(4), e18-e18.

Gao, F., Chen, C., Arab, D. A., Du, Z., He, Y., & Ho, S. Y. (2019). EasyCodeML: A visual tool for analysis of selection using CodeML. Ecology and evolution, 9(7), 3891-3898.

Greiner, S., Lehwark, P., & Bock, R. (2019). OrganellarGenomeDRAW (OGDRAW) version 1.3. 1: expanded toolkit for the graphical visualization of organellar genomes. Nucleic acids research, 47(W1), W59-W64.

Hendrickson, S. L., Bleiweiss, R., Matheus, J. C., de Matheus, L. S., Jácome, N. L., & Pavez, E. (2003). Low genetic variability in the geographically widespread Andean Condor. Condor, 105(1), 1–12.

Jin, J. J., Yu, W. B., Yang, J. B., Song, Y., Depamphilis, C. W., Yi, T. S., & Li, D. Z. (2020). GetOrganelle: a fast and versatile toolkit for accurate de novo assembly of organelle genomes. Genome biology, 21(1), 1-31.

Katoh, K., & Standley, D. M. (2013). MAFFT multiple sequence alignment software version 7: improvements in performance and usability. Molecular biology and evolution, 30(4), 772-780.

Langmead, B., & Salzberg, S. L. (2012). Fast gapped-read alignment with Bowtie 2. Nature methods, 9(4), 357.

Li, H. (2018). Minimap2: pairwise alignment for nucleotide sequences. Bioinformatics, 34(18), 3094-3100.

Wick, R. R., Judd, L. M., Gorrie, C. L., & Holt, K. E. (2017). Unicycler: resolving bacterial genome assemblies from short and long sequencing reads. PLoS computational biology, 13(6), e1005595.
